# A Humanized Monoclonal Antibody Targeting Extracellular Nicotinamide Phosphoribosyltransferase Prevents Aggressive Prostate Cancer Progression

**DOI:** 10.3390/ph14121322

**Published:** 2021-12-17

**Authors:** Belinda L. Sun, Lin Tang, Xiaoguang Sun, Alexander N. Garcia, Sara M. Camp, Edwin Posadas, Anne E. Cress, Joe G. N. Garcia

**Affiliations:** 1 Department of Pathology, College of Medicine, University of Arizona Health Sciences, Tucson, AZ 85719, USA; bsun@arizona.edu; 2 Department of Medicine, College of Medicine, University of Arizona Health Sciences, Tucson, AZ 85719, USA; ltang@arizona.edu (L.T.); xgsun@arizona.edu (X.S.); saramcamp@arizona.edu (S.M.C.); 3 Department of Radiation Oncology, College of Medicine, University of Arizona Health Sciences, Tucson, AZ 85719, USA; alexandernathanielgarcia@gmail.com; 4 Department of Medicine, Cedar Sinai Health Sciences, Los Angeles, CA 90048, USA; edwin.posadas@cshs.org; 5 Department of Cellular and Molecular Medicine, College of Medicine, University of Arizona Health Sciences, Tucson, AZ 85719, USA; cress@arizona.edu

**Keywords:** prostate cancer, extracellular nicotinamide phosphoribosyltransferase (eNAMPT), orthotopic xenograft mouse model, castration-resistant prostate cancer (CRPC), eNAMPT-neutralizing monoclonal antibody (ALT-100), damage-associated molecular pattern protein

## Abstract

Prostate cancer (PCa) is the major cause of cancer-related death in males; however, effective treatments to prevent aggressive progression remain an unmet need. We have previously demonstrated that secreted extracellular nicotinamide phosphoribosyltransferase (eNAMPT) is a multifunctional innate immunity regulator that promotes PCa invasion. In the current study, we further investigate the therapeutic effects of an eNAMPT-neutralizing humanized monoclonal antibody (ALT-100 mAb) in preclinical PCa orthotopic xenograft models. We utilized human aggressive PCa cells (DU145 or PC3) for prostate implantation in SCID mice receiving weekly intraperitoneal injections of either ALT-100 mAb or IgG/PBS (control) for 12 weeks. Prostatic tumors and solid organs were examined for tumor growth, invasion, and metastasis and for biochemical and immunohistochemistry evidence of NFκB activation. ALT-100 mAb treatment significantly improved overall survival of SCID mice implanted with human PCa orthotopic prostate xenografts while inducing tumor necrosis, decreasing PCa proliferation and reducing local invasion and distal metastases. The ALT-100 mAb inhibits NFκB phosphorylation and signaling in PCa cells both in vitro and in vivo. This study demonstrates that eNAMPT neutralization effectively prevents human PCa aggressive progression in preclinical models, indicating its high potential to directly address the unmet need for an effective targeted therapy for patients with aggressive PCa.

## 1. Introduction

Prostate cancer (PCa) is the most common cancer and the second leading cause of cancer-related death in men in the United States [1]. Lethal PCa evolves through disease progression [2], mostly from androgen-driven adenocarcinomas to castration-resistant prostate cancer (CRCP), with reduced responsiveness to current standard androgen deprivation therapy and chemotherapy [3,4,5]. The overall 5-year survival rate declines from 98% to 30% in progressed metastatic PCa [6,7]. Thus, there is an urgent unmet need to develop therapeutic approaches to treat PCa aggressive progression and reduce PCa lethality.

Current mechanistic concepts for PCa transition to aggressive, metastatic CRPC have highlighted the involvement of a hypoxic tumor environment [8], inflamed peri-prostatic adipose tissues [9], and inflammatory signaling pathways [10] that activate androgen receptors and promote PCa progression. We previously demonstrated that extracellular nicotinamide phosphoribosyltransferase (eNAMPT) is a damage-associated molecular pattern protein (DAMP) with the capacity to uniquely bind and activate Toll-like receptor 4 (TLR4) [11,12], thereby markedly triggering NFκB-dependent signaling pathways to promote cell immune response, survival, and growth [12,13]. TLR4 is expressed on PCa cells, tumor-infiltrating lymphocytes, and macrophages and has been strongly linked to PCa tumorigenesis and progression including survival, migration, and invasion [14,15]. We have previously demonstrated eNAMPT is a highly druggable target that is expressed in human invasive PCa cells including aggressive CRPC cells with an increased release of eNAMPT, a critical upstream DAMP that dramatically enhances tumor invasion [16]. In addition, we have shown that *NAMPT* transcription and eNAMPT secretion are potently stimulated by hypoxia in an HIF-2α-dependent manner [17], potentially influencing the PCa tumor microenvironment. These pathobiological functions support eNAMPT as a clinically relevant therapeutic target with the potential to prevent PCa lethal progression. The present study is designed to extend our prior report that a polyclonal eNAMPT-neutralizing antibody prevents PCa invasion into diaphragmatic muscle tissues in animal models in vivo [16]. In the present study, we utilized a humanized eNAMPT-neutralizing monoclonal antibody (ALT-100 mAb) in preclinical human PCa orthotopic xenograft animal models to further validate a contributory role for eNAMPT in PCa local invasion and distant metastasis. Human PCa cells, DU145 or PC3, were injected into the prostate of adult male SCID mice to generate orthotopic xenografts, with mice receiving an intraperitoneal injection of either an IgG vehicle or the ALT-100 mAb. We found the eNAMPT-neutralizing ALT-100 mAb to significantly increase survival of SCID mice with human PCa orthotopic xenografts and to significantly inhibit PCa cell proliferation, invasion, and metastases. These studies validate eNAMPT as a highly druggable therapeutic target and ALT-100 mAb as a potential therapeutic strategy to directly address the unmet need for novel and effective treatments to limit PCa lethality.

## 2. Results

### 2.1. The eNAMPT-Neutralizing ALT-100 mAb Significantly Increases Survival of SCID Mice with Human PCa Orthotopic Xenografts

We have developed a humanized anti-eNAMPT monoclonal antibody (ALT-100) derived from murine hydridomas (Abpro, Boston, MA, USA) with subsequent humanization (Fusion Antibodies, Belfast, UK). ALT-100 mAb was identified after screening with in vitro endothelial cell electrical resistance assays and NFκB activation biochemical assays and in vivo preclinical murine lung injury models [18]. The eNAMPT mAb exhibits high eNAMPT binding affinity (K_d_ of 6.33 nM) with pharmacokinetic studies demonstrating a T^1/2^ half-life of 12–14 days in rats (Supplemental Data Figures S1 and S2).

**Figure 1:**
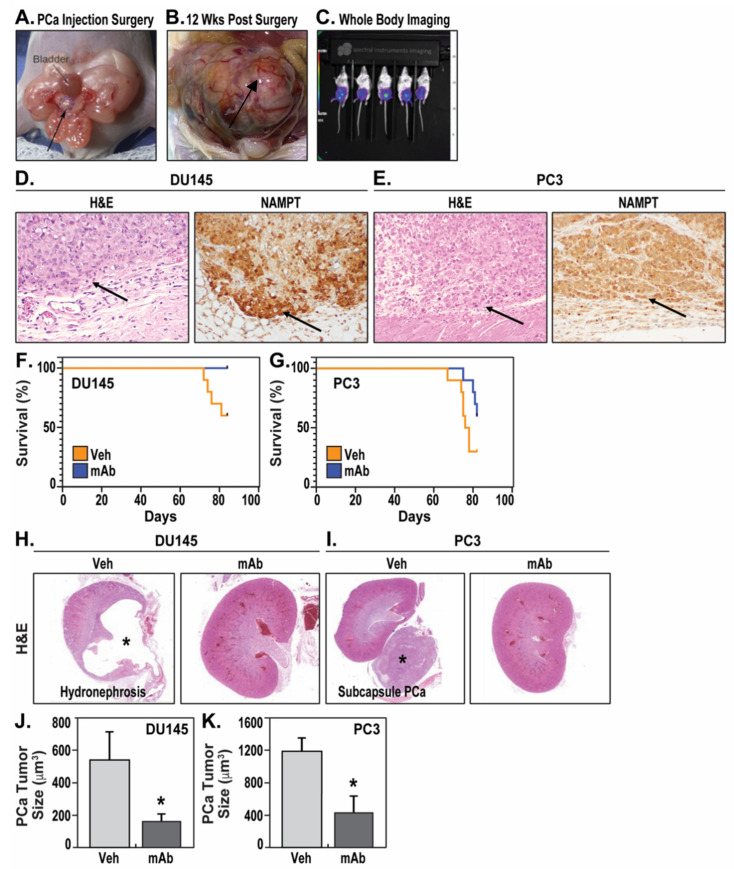
The eNAMPT-neutralizing mAb, ALT-100, increases survival of SCID mice with human PCa orthotopic xenografts. (**A**): DU145^luc^ or PC3^luc^ cells were implanted into the anterior lobe of prostate (arrow/circle) of SCID mice. (**B**): PCa tumors grew into larger prostate masses (arrow) at 12 weeks after implantation. (**C**): Live mice were whole body imaged to monitor tumor growth and location (purple shading indicates PCa tumor in the prostate). (**D**): Staining showed DU145 xenograft (H&E, 200×, arrow) and strong NAMPT expression (IHC, 200×, brown color). H&E arrow shows DU145 xenograft growing in extracapsular soft tissues of the prostate. NAMPT arrow shows DU145 xenograft with strongly expressed NAMPT staining in brown color. (**E**): Staining showed PC3 xenograft (H&E, 200×, arrow) and strong NAMPT expression (IHC, 200×, brown color). H&E arrow shows PC3 xenograft invading into capsular tissue of the prostate. NAMPT arrow shows PC3 xenograft with strongly expressed NAMPT staining in brown color. (**F**): Survival curve of mice with DU145 orthotopic xenograft showed 100% survival in mAb-treated group (mAb) compared to 60% survival in vehicle-treated group (veh) at the study endpoint of 12 weeks (84 days). (**G**): Survival curve of mice with PC3 orthotopic xenograft showed 60% survival in mAb-treated group compared to 30% survival in vehicle-treated group at the study endpoint of 12 weeks. (**H**): H&E staining showing dramatic hydronephropathy with dilated renal pelvis (*) in vehicle-treated DU145 PCa-exposed mice, versus H&E staining of kidneys from mice with DU145 xenografts receiving weekly ALT-100 mAb treatment. (**I**): Subcapsular PC3 metastases grew into a large mass () approximating the size of a kidney, versus unremarkable kidneys of mice with PC3 xenografts receiving weekly ALT-100 mAb treatment. (**J**): The sizes of prostate DU145 tumors were significantly smaller in ALT-100 mAb-treated group than that in IgG vehicle-treated group, * *p* < 0.05. (**K**): The sizes of prostate PC3 tumors were significantly smaller in ALT-100 mAb-treated group than those in IgG vehicle-treated group, * *p* < 0.05.

DU145 and PC3 are human-aggressive PCa cells and often utilized for PCa studies examining the efficacy of various therapeutics. We tested the therapeutic efficacy of the ALT-100 mAb in human PCa orthotopic xenograft mouse models in which human DU145 or PC3 cells were implanted into the prostate of SCID male mice as primary tumor models of PCa [19] (Figure 1A–C). Growth of the primary tumor in the prostate was accompanied by subsequent invasion of adjacent structures and metastasis to distal organs.

DU145 xenografts (Figure 1D) and PC3 xenografts (Figure 1E) both exhibited high NAMPT expression in vivo. Mortality in DU145 orthotopic xenograft mice began to increase 10 weeks after DU145 cell implantation with a 40% mortality at 12 weeks. In contrast, ALT-100-treated DU145 mice experienced 100% survival at the study endpoint of 12 weeks (one mouse expired due to general anesthesia for imaging study in week 12) (Figure 1F). Both log-rank (Mantel–Cox) test (*p* = 0.029) and Gehan–Breslow–Wilcoxon test (*p* = 0.03) showed significant differences in survival probability between IgG-treated and ALT-100-treated DU145 mice (*p* < 0.05). These data suggest that mice receiving the eNAMPT-neutralizing mAb exhibit significantly improved overall survival with DU145 orthotopic xenografts.

In PC3 orthotopic xenograft models, vehicle IgG-treated mice began to expire at 9 weeks after PC3 implantation, with survival rates of 70% at 11 weeks and 30% at 12 weeks. In contrast, weekly delivery of the ALT-100 mAb delayed the onset of deaths to 10 weeks with 90% survival rate at 11 weeks and 60% survival at 12 weeks (Figure 1G). Gehan-Breslow–Wilcoxon test, which places greater weight on deaths at early time points (9 weeks and 10 weeks), showed significant differences in survival probability between the IgG-treated and ALT-100-treated group (*p* = 0.043). The log-rank (Mantel–Cox) test, which places equal weight on all time points, trended toward significant differences in survival probability but did not achieve significance (*p* = 0.07). These results indicate the ALT-100 mAb provides survival benefit in both DU145 and PC3 orthotopic xenograft models.

Pathologic necropsies identified the cause of death in vehicle IgG-treated DU145 orthotopic xenograft mice as severe bilateral kidney hydronephrosis secondary to tumor-induced urinary obstruction (Figure 1H). Bilateral hydronephrosis was also observed in 60% of surviving mice at the study endpoint of 12 weeks in the IgG-treated group. In contrast, only one ALT-100-treated DU145 mouse showed unilateral hydronephrosis, whereas the remaining treated mice failed to display gross renal pathology. In contrast, the cause of death in IgG-treated PC3 orthotopic xenograft mice was related to tumor burden rather than urinary obstruction without evidence of tumor-induced urinary tract obstruction or hydronephrosis. Instead, vehicle IgG-treated PC3 mice exhibited large pelvic cavity masses, including large subcapsular kidney masses (Figure 1I), indicating high tumor burden systemically rather than local obstruction as the major causes of death. Measurements of tumor size showed PC3 tumors grew to nearly double the size of DU145 tumors in the same 12-week period, but both DU145 (Figure 1J) and PC3 (Figure 1K) tumors significantly decreased in size in ALT-100 mAb-treated groups compared with control IgG vehicle-treated groups.

### 2.2. The eNAMPT-Neutralizing ALT-100 mAb Significantly Inhibits PCa Proliferation

**Figure 2:**
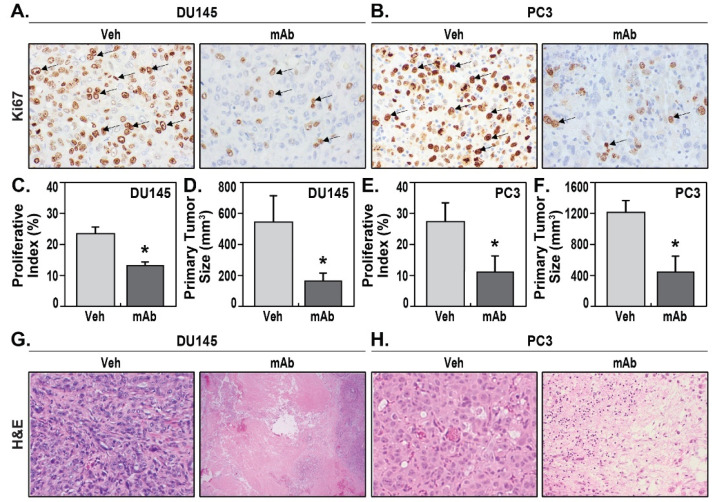
The eNAMPT-neutralizing mAb, ALT-100, inhibits PCa proliferation. (**A**): IHC for proliferation index Ki67 showed much lower percentage of Ki67-positive DU145 cells (arrows) in mAb-treated compared to vehicle-treated xenografts. (**B**): IHC for Ki67 showed much lower percentage of positive PC3 cells (arrows) in mAb-treated compared to vehicle-treated xenografts. (**C**): Statistic analysis on DU145 xenografts showed Ki67 proliferation index was significantly lower in mAb-treated group than in the vehicle treated group (* *p* < 0.05). (**D**): Measurement on DU145 xenografts showed significantly smaller tumor size in mAb-treated group than in the vehicle-treated group (* *p* < 0.05). (**E**): Statistic analysis on PC3 xenografts showed Ki67 proliferation index was significantly lower in mAb-treated group than in the vehicle-treated group (* *p* < 0.05). (**F**): Measurement on PC3 xenografts showed significantly smaller tumor size in ALT-100 mAb-treated group than vehicle-treated group (* *p* < 0.05). (**G**): Solid DU145 tumor growth in vehicle-treated xenografts (H&E, 200×), whereas ALT-100 mAb treatment induced extensive tumor necrosis in DU145 xenografts (H&E, 200×). (**H**): Solid PC3 tumor growth in vehicle-treated xenografts (H&E, 200×), whereas ALT-100 mAb treatment induced tumor necrosis in PC3 xenografts (H&E, 200×).

The PCa xenografts were measured for tumor size via microscopic image analysis, and PCa cell proliferation was assessed via immunohistochemical examination of the proliferative index, Ki67. DU145 xenografts in prostate displayed moderate proliferation rates averaging 23.3% ± 1.9% Ki67 proliferative index in vehicle IgG-treated mice (Figure 2A,C). Treatment with the ALT-100 mAb significantly reduced DU145 prostate tumor proliferation to 12.9% ± 1.1% Ki67 proliferative index (*p* < 0.05) (Figure 2A,C). Consistent with these results, DU145 xenografts showed significantly smaller tumor volume in ALT-100-treated DU145 mice, averaging 165.2 ± 49.3 mm^3^ compared to the average of 538.8 ± 170.6 mm^3^ in vehicle IgG-treated mice (Figure 2D).

PC3 is considered as an aggressive castration-resistant PCa cell, and compared to DU145 xenografts, PC3 xenograft exhibited a relatively higher proliferative index of 27.3% ± 6.1% (Figure 2B,E). Treatment with the ALT-100 mAb significantly decreased the proliferation rate to an average of 13.3% ± 5.7% in treated group (Figure 2B,E). Similarly, the tumor volume of viable PC3 xenografts in ALT-100-treated group averaged 418.03 ± 219 mm^3^, which was significantly smaller (*p* < 0.05) than the average of 1197.09 ± 518 mm^3^ in the IgG-treated group (Figure 2F). ALT-100-treatment induced extensive histologic evidence of tumor necrosis in treated group in both DU145 (Figure 2G) and PC3 xenografts (Figure 2H). The tumor necrotic areas were not counted as viable tumor volumes under microscopic measurement. The experiments for each group are summarized in Table 1.

### 2.3. The eNAMPT-Neutralizing ALT-100 mAb Significantly Inhibits PCa Invasion and Metastasis

**Figure 3:**
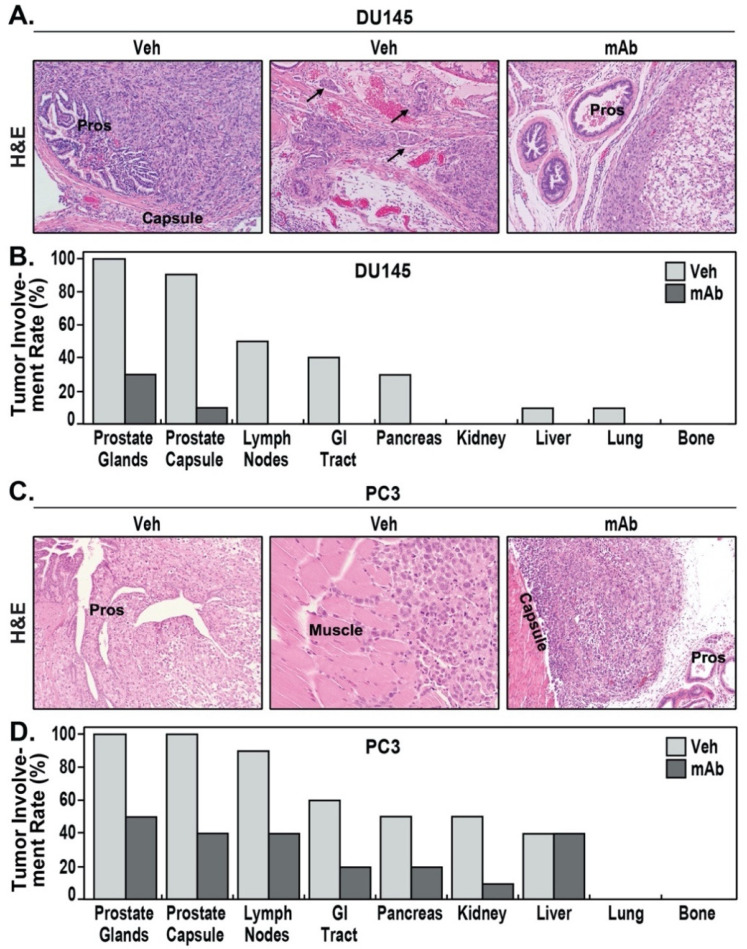
The eNAMPT-neutralizing mAb, ALT-100, prevents PCa invasion and metastasis. (**A**): DU145 xenograft invaded prostate glands (pros) and capsule in vehicle IgG-treated mice (**left panel**), and lymphovascular invasion (arrows, **middle panel**) was easily identified in DU145 xenografts in vehicle IgG-treated mice. In mAb-treated mice (**right panel**), DU145 xenografts appeared to push prostate glands (pro) without infiltration and lymphovascular invasion. (**B**): Percentage of mice with organ invasion and metastasis in vehicle-treated and mAb-treated DU145 orthotopic xenografts. No distant metastasis was observed in mAb-treated group. (**C**): PC3 xenograft extensively invaded prostate glands (pros) (**left panel**) and locally spread and directly invaded into pelvic skeletal muscle (**middle panel**). In mAb-treated mice (**right panel**), PC3 grew with a defined border without extensive invasion into the capsule and prostate glands (pros). (**D**): Percentage of mice with organ invasion and metastasis in vehicle-treated and mAb-treated PC3 orthotopic xenografts. Lower metastasis percentages were observed in mAb-treated group compared to vehicle-treated group. (**A**,**C**), H&E 200×.

IgG-treated DU145 orthotopic xenografts grew locally into large prostate tumor masses. DU145 PCa cells were cohesive but exhibited an infiltrative border with invasion of adjacent prostate glands and capsules (Figure 3A) and clear evidence of lymphovascular invasion (Figure 3A). IgG-treated DU145 primary tumors exhibited 100% invasion into prostate glands, 90% capsular invasion, and 50% lymphovascular invasion (Figure 3B). In contrast, the xenografts in the ALT-100-treated group showed a well-circumscribed border without obvious tumor infiltration or lymphovascular invasion (Figure 3A). Only 30% of ALT-100-treated primary tumors showed tumor invasion into prostate glands, and only 10% had focal capsular invasion. DU145 xenografts displayed moderate metastatic potential with five of ten IgG-treated mice (50%) showing multiple (ranging from one to four) pelvic lymph nodes positive for metastatic DU145 (Figure 3B). In the five mice with lymph node metastasis, four (40%) also showed intestinal tract tumor involvement, three (30%) showed pancreatic metastasis, one (10%) showed liver metastasis, and one (10%) showed lung metastasis. No metastasis was identified in other organs including kidney, heart, or bone. In contrast, 100% of ALT-100-treated mice were free of lymph node or distal organ metastases (Figure 3B).

Vehicle IgG-treated PC3 orthotopic xenografts had higher metastatic potential compared to DU145 cells, grew locally with marked border infiltration, and experienced extensive local spread and prostate gland destruction (Figure 3C). PC3 tumors extended beyond the prostate and directly invaded pelvic wall skeletal muscle (Figure 3C). IgG-treated primary PC3 tumors showed 100% prostate gland extension (Figure 3D), 100% extensive capsular invasion, and 90% pelvic lymph node involvement (ranging from 2 to 7 nodes). In contrast, PC3 orthotopic xenografts in the ALT-100-treated group grew as circumscribed nodules with much less invasive borders (Figure 3C). Prostate gland and local invasion were observed in 50% and 40% of ALT-100-treated mice, compared to 100% in IgG-treated mice.

Distal metastases were prominent in vehicle IgG-treated PC3 orthotopic xenografts with 90% lymph nodes involvement (9 of 10 mice), 60% intestinal tract involvement, 50% pancreatic involvement, 50% kidney involvement, and 40% liver involvement. In contrast, ALT-100 mAb-treated PC3 orthotopic xenografts exhibited only 40% lymph nodes involvement (4 of 10 mice), 40% intestinal tract involvement, 20% pancreatic involvement, 10% kidney involvement, and 40% liver involvement (Figure 3D). While both ALT-100-treated and vehicle IgG-treated groups exhibited 40% liver metastatic involvement, sizes of the metastases differed significantly with ALT-100 mAb-treated liver tumors significantly smaller (averaging 63.64 ± 5.3 mm^3^) compared to average liver tumor size of 452.12 ± 206.03 mm^3^ in vehicle IgG-treated mice. Metastases were not identified in lung, heart, or bone tissues from either group.

### 2.4. The eNAMPT-Neutralizing ALT-100 mAb Inhibits NFκB Signaling in Human PCa Cells

**Figure 4:**
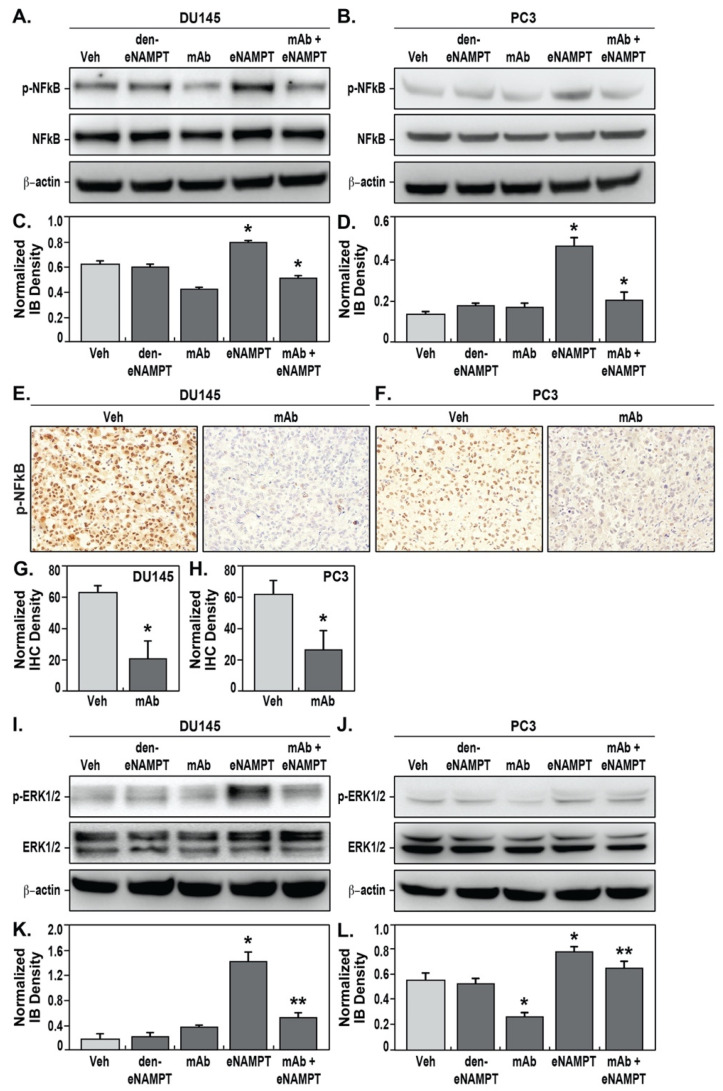
The eNAMPT-neutralizing ALT-100 mAb inhibits NFκB phosphorylation in PCa cells and xenografts. DU145 cells were exposed to vehicle (PBS), denatured eNAMPT (den-eNAMPT), anti-eNAMPT mAb (mAb), eNAMPT, or mAb mixed with eNAMPT. Immunoblot for phosphor-NFkB p65 (p-NFkB), total NFkB, and internal control beta-actin showed that mAb-treatment significantly decreased eNAMPT-induced NFκB phosphorylation in DU145 cells (**A**) and PC3 cells (**B**). Immunoblot density measurement showed that mAb treatment significantly decreased eNAMPT-induced NFκB phosphorylation level in DU145 cells (**C**) and PC3 cells (**D**) (* *p* < 0.05). IHC for phosphor-NFκB p65 (p-NFκB) showed high levels of NFκB phosphorylation (brown) in vehicle-treated xenografts, whereas the level was markedly decreased in mAb-treated DU145 (**E**) and PC3 xenografts (**F**). Measurement of IHC density showed that phosphor-NFκB p65 (p-NFκB) was significantly lower in mAb-treated DU145 (**G**) and PC3 xenografts (**H**) compared to vehicle IgG-treated mice (* *p* < 0.05). 100x. Immunoblots for phosphor-ERK1/2 (p-ERK1/2), total ERK1/2, and internal control beta-actin showed that mAb-treatment significantly decreased eNAMPT-induced ERK1/2 phosphorylation in DU145 (**I**,**K**) and PC3 cells (**J**,**L**) quantified by immunoblot densitometric measurements (*,** *p* < 0.05).

We previously demonstrated that eNAMPT-mediated signaling occurs via the ligation of TLR4 [12,13] and triggering of NFκB activation, an inflammatory pathway well studied in PCa that is suggested to promote PCa survival, invasion, metastasis, and chemoresistance [20]. We challenged DU145 (Figure 4A) and PC3 cells in vitro (Figure 4B) with eNAMPT, thereby eliciting significant NFκB signaling and NFκB phosphorylation, which was not observed in cells exposed to denatured eNAMPT. PCa cell exposure to ALT-100 mAb abolished the eNAMPT-mediated NFκB activation response (Figure 4A–D).

In the PCa orthotopic xenograft models, the effects of ALT-100 mAb treatment on NFκB activation were studied via immunohistochemical staining for phosphor-NFκB in PCa tumors. In IgG-treated groups, DU145 and PC3 tumors both showed strong nuclear staining for phosphor-NFκB (Figure 4E,F). In ALT-100 mAb-treated mice, the levels of phosphor-NFκB in the DU145 and PC3 xenografts were markedly decreased in vivo (Figure 4E,F) confirming the involvement of the eNAMPT/TLR4/NFκB signaling pathway in these in vivo PCa xenograft tumor models. We further explored potential eNAMPT-induced extracellular signal-regulated kinase (ERK) signaling, a known NFκB cross-talking pathway involving cell proliferation and tumorigenesis in DU145 and PC3 cells. These studies demonstrated that eNAMPT significantly stimulates ERK1/2 phosphorylation in both DU145 (Figure 4I,K) and PC3 cells (Figure 4J,L), which was reduced in cells treated with the eNAMPT- neutralizing ALT-100 mAb.

## 3. Discussion

This study was designed to address the urgent and unmet need for PCa therapies that effectively combat aggressive PCa disease. Similar to studies targeting eNAMPT in preclinical models of radiation pneumonitis [21] and pulmonary hypertension [22], we evaluated the therapeutic effects of an eNAMPT-neutralizing humanized monoclonal antibody, ALT-100, in two human PCa orthotopic xenograft SCID mice models with implantation of aggressive PCa cells, DU145 or PC3, representing both PTEN-positive (DU145) and PTEN-negative (PC3) tumor subtypes. In these models, compared to the IgG-treated group, mice weekly receiving the eNAMPT-neutralizing ALT-100 mAb exhibited improved overall survival, decreased PCa cell proliferation, decreased tumor local invasion, and limited distant metastases. At the molecular level, ALT-100 treatment prevents eNAMPT-mediated NFκB signaling pathway activation and NFκB phosphorylation in PCa tumors. This study demonstrates that eNAMPT-neutralizing antibody therapy has significant potential as an effective therapy for the treatment of aggressive PCa including castration-resistant, PTEN-positive, and PTEN-negative PCa.

PCa cell heterogeneity [23] often results in phenotypical differences within or among PCa individuals. In this study, we observed better outcomes in ALT-100-treated DU145 xenograft mice models compared to PC3 xenografts, findings that potentially reflect PCa heterogeneity in these two often-studied standard aggressive PCa cells. DU145 is a human PCa cell line derived from brain metastasis of a 69-year-old Caucasian male with grade II (low-grade) primary PCa and is androgen receptor-positive but hormone-insensitive with no expression of prostate-specific antigen (PSA). PC3 is a human castration-resistant PCa derived from the bone metastasis of a 62-year-old Caucasian male with grade IV (high-grade) primary PCa and is androgen receptor-negative. Both DU145 and PC3 cells intrinsically highly express eNAMPT with DU145 tumors demonstrating high therapeutic responsiveness to ALT-100 with complete prevention of vascular invasion and distal metastases and 100% cancer-related survival. In contrast, PC3 tumors exhibited only partial ALT-100 responsiveness with less improved overall survival. Although still significant, the ALT-100 mAb was less effective in PC3 tumors in inhibiting invasion and metastasis at the 12-week study endpoint. Further investigation is required to integrate the relative role eNAMPT/TLR4 signaling in different PCa tumors, including potential differences in *NAMPT* polymorphisms between the two PCa cell types. These studies will inform the potential tailoring of the ALT-100 mAb treatment for likely responders to this eNAMPT-targeted therapy.

Lethal PCa progression evolves through several disease stages [2] with androgen receptors [24], oncogenes, and the tumor microenvironment [25] serving as major mechanisms that lead to PCa progression. Under certain selective pressure or an altered microenvironment, PCa evolves from androgen-driven to autocrine- or paracrine-driven cancers. This transition often signals tumor invasion and dissemination and eventually leads to an “autonomous stage” of castration-resistant PCa, which is incurable with current standard therapies. Current mechanistic concepts for this PCa transition to aggressive, metastatic CRPC have highlighted the involvement of a hypoxic tumor environment, inflamed peri-prostatic adipose tissues, and inflammatory signaling pathways that activate androgen receptors and promote PCa progression. It is well known that the hypoxic tumor microenvironment results in hypoxia-induced transcription factors (HIF-1α, HIF-2α)-mediated production of locally produced chemokines/cytokines [26,27,28,29,30] emanating from normal prostate epithelial cells, periprostatic adipocytes, and PCa cells. Thus, a novel therapy that targets inflammatory signaling pathways may represent a potent mechanism to prevent PCa progression and to reduce PCa lethality. We speculate that eNAMPT activation of the TLR4/NFkB-dependent inflammatory cascade [11,12] contributes to the progression of CRPC [10]. We reported that *NAMPT* transcription and eNAMPT secretion are highly upregulated by hypoxia via HIF-2α signaling [17] and tumor-relevant growth factors [16]. eNAMPT secretion is basally augmented in CRPC cells (PC3) compared to normal prostate epithelium [16]. We identified increased NAMPT staining in human PCa tissues with plasma eNAMPT levels significantly correlated with the degree of PCa invasiveness in men with PCa [16]. We speculate that aggressive PCa, capsule-invading metastatic disease involves a hypoxic tumor microenvironment resulting in HIF-2α-increased eNAMPT secretion by epithelial cells [17], periprostatic adipocytes [31], PCa cells [16] and tumor-supporting tissue M2 macrophages whose generation we have shown is eNAMPT-enabled [32]. Functioning as a DAMP, eNAMPT triggers TLR4/NFκB inflammatory signaling to locally produce chemokines/cytokines and growth factors that increase PCa invasiveness [16]. This study further confirms the utility of targeting the autocrine/paracrine mediators as potentially potent mechanisms to prevent PCa aggressive progression with the eNAMPT-targeted ALT-100 mAb significantly dampening inflammatory cascade activation and preventing PCa aggressive progression in therapy in human PCa orthotopic xenograft models.

NAMPT’s intracellular enzymatic activity (iNAMPT) is the essential rate-limiting step in nicotinamide adenine dinucleotide (NAD) biosynthesis, acting via a salvage pathway that is critical for maintaining human cell survival under hypoxia or nutritional depletion [33]. NAMPT-mediated NAD biosynthesis controls the functions of mammalian sirtuin family members and NAD-consuming enzymes such as PARPs, in each subcellular compartment, and therefore involving a variety of critical biological processes, including cell metabolism, survival, and stress response [33]. Elevated iNAMPT tumor expression is associated with poor overall survival in variable cancers [34]. Currently, several phase I clinical trials are completed or in progress utilizing intracellular iNAMPT enzymatic inhibitors (GMX-1776/CHS-828, APO-866/Daporinad/FK866) as anti-cancer therapies; however, limited therapeutic benefit and significant dose-limiting toxicities were observed [35,36], likely due to non-specific inhibition of iNAMPT, an essential metabolic enzyme [37].

As knowledge on NAMPT involvement in cancer pathobiology is continuously evolving [38], we have focused on the role of the novel DAMP, eNAMPT, a key immunity and metabolic regulator involving diverse physiological and pathological processes [21,22,34,38,39,40] and a highly druggable therapeutic target, in preventing aggressive PCa progression [12]. In this study, although we have not directly investigated TLR4 in PCa, we observed clear evidence that eNAMPT stimulates NFκB phosphorylation and neutralizing eNAMPT treatment inhibits NFκB activation in PCa cells. These findings at least partially explain the therapeutic effects of eNAMPT-neutralizing treatment in Pca cells, although the comprehensive signaling pathways and cross-interactions need to be further studied.

In summary, the present study extends our prior report that a polyclonal eNAMPT-neutralizing antibody prevents PCa invasion in a PCa diaphragm invasion animal model in vivo [16] and strongly supports eNAMPT as a clinically relevant, highly druggable therapeutic target. The humanized eNAMPT-neutralizing monoclonal antibody (ALT-100 mAb) is a potential therapeutic strategy to directly address the unmet need for novel and effective treatments to prevent PCa progression and limit PCa lethality.

## 4. Materials and Methods

### 4.1. eNAMPT-Neutralizing Humanized mAb ALT-100

ALT-100 mAb was provided by Aqualung Therapeutics Corporation, Tucson, AZ. Details of the generation of this humanized eNAMPT-neutralizing mAb from murine mAbs have been previously reported [16,18]. Mice receiving this mAb on a weekly basis for 12 weeks exhibited no discernable toxicity with mAb treatment as previously reported in hypoxia/Sugen-exposed mice [22] and radiation-exposed mice [21].

### 4.2. Human PCa Cell Lines and Cell Cultures

DU145 (ATCC^®^ HTB-81™, Manassas, VA, USA) and PC3 (ATCC^®^ CRL-1435™, Manassas, VA, USA) were purchased from American Type Culture Collection (ATCC, Manassas, VA, USA), Manassas, Virginia. The cell lines were authenticated by using the short tandem repeat (STR) profiling, showing a >85% match to the data bank provided, and thus they were deemed authentic. All populations tested negative for mycoplasma and murine viruses. DU145 and PC3 cells were grown and maintained in RPMI-1640 medium containing antibiotics, 2 mM of L-glutamine and 10% fetal bovine serum. Cells were grown in T-25 cm3 flasks to an adherent monolayer culture in a humidified atmosphere of 5% CO_2_ at 37° and treated with PBS (control), inactivated eNAMPT (boiled for 5 min) (3 ug/mL), ALT-100 (10 ug/mL), eNAMPT (3 ug/mL), and eNAMPT (3 ug/mL) + ALT-100 (10 ug/mL). All experiments were repeated at least 3 times.

DU145^Luc^ and PC3^Luc^ cells expressing a pLazarus retroviral construct (pGL4.5(luc2/cmv/neo), SnapGene, San Diego, CA, USA) containing luciferase were constructed by using the FuGene Transfection Reagent (Fugent LLC, Middleton, WI, USA) according to the manufacturer’s instructions and positively selected by treatment of G418 (geneticin, 200 ug/mL, InvivoGen, San Diego, CA, USA) 36 h after transfection. The luciferase-labeled DU145^Luc^ and PC3^Luc^ cells were injected into the prostate of male SCID mice, allowing us to monitor tumor growth by using whole body imaging (Lago X-Spectral Instruments Imaging, Accela S.R.O., Czech Republic).

### 4.3. PCa Orthotopic Xenograft Mouse Models

DU145 ^Luc^ or PC3^Luc^ (1 × 10^5^ in 20 μL of PBS) were injected into the anterior lobe of prostate of 7- to 10-week-old male SCID (C.B-Igh-1b/IcrTac-Prkdcscid) mice. After being fully anesthetized in isoflurane chamber, SCID mice were placed in a sterile procedure hood and disinfected at lower abdominal region with a Betadine solution. A low midline incision (3–4 mm) was made at the lower abdomen, and the bladder was gently lifted to expose anterior (ventral) lobe of prostate. Injection of 20 µl cell solution was performed using microinjection syringe and a small bubble of injected solution was observed in prostate (Figure 1A), as described previously [41]. After surgery, SCID mice were whole body imaged for luciferase activity to confirm tumor cell location and survival in prostate.

Seven days after injection of PCa cells into the prostate, SCID mice with successful tumor implantation were initially grouped using a randomized table and received intraperitoneal injection 1×/week with either vehicle (non-specific human IgG_4_ 0.4 mg/kg in PBS, i.e., the vehicle IgG-treated group, *n* = 10) or with ALT-100 (0.4 mg/kg in PBS, i.e., the mAb-treated group, *n* = 10). Tumor growth was monitored weekly by using whole body imaging and manual palpation, and the tumor size was estimated by using a digital caliper measuring the diameter of palpable mass. On achieving the study endpoint at 12 weeks, the animals were anesthetized, and prostate, bladder, kidneys, adrenal glands, liver, spleen, diaphragm, heart, lungs, periaortic and pelvic lymph nodes, brain, spine, and skeletal bones were collected and processed for pathologic examination. Tissues were fixed in formalin and processed for microscopic examination and measurement. The primary tumor with prostate tissue were examined for tumor size, morphology, necrosis, and invasion. All other organs were processed for pathologic examination for metastasis. If death occurred during the study, a necropsy was performed, and all of the organs were examined grossly and microscopically to determine the cause of death, and the tumor size, local invasion, and distal metastasis were analyzed by pathologic examination as well.

### 4.4. Detection of NFκB Activation by Western Blot

As we described previously [12], protein extracts from PC3 and DU145 cells were separated by 10% SDS-PAGE, transferred to nitrocellulose membranes (100 V for 1.5 h), and immunoreacted with a rabbit anti-human phosphorylated-NFκB polyclonal antibody (1:1000, Bethyl Laboratories, Inc, Montgomery, TX, USA), total-NFκB (1:1000, Bethyl Laboratories, Inc, Montgomery, TX, USA) or mouse anti-human β-actin monoclonal antibody (1:1000, Bethyl Laboratories, Inc, Montgomery, TX, USA). Immunoreactive proteins were detected with the enhanced chemiluminescent detection system according to the manufacturer’s directions (Amersham, Little Chalfont, UK). Intensities of immunoreactive protein bands were quantified using ImageQuant software (Molecular Dynamics, Sunnyvale, CA, USA). All experiments were repeated a minimum of three times.

### 4.5. Immunohistochemistry and Analysis

Immunohistochemistry for phosphor-NFκB (1:100, Bethyl Laboratories, Inc, Montgomery, TX, USA) was performed on primary tumors, as described previously [16]. Paraffin-embedded tissue blocks of PCa and benign prostate tissues were processed to 4-micrometer paraffin tissue sections. Three representative tissue blocks and six sequential tissue sections per tissue block were processed for each mouse. After rehydration and serum blocking, the paraffin sections were sequentially incubated with a rabbit anti-human phosphor-NFκB polyclonal antibody with a dilution of 1:100 (Bethyl Laboratories, Inc, Montgomery, TX, USA) or anti-human Ki67 monoclonal antibody (1:100, rabbit monoclonal antibody, clone 30-9, Ventana Medical Systems, Inc., Tucson, AZ, USA) HRP-conjugated ABC kit (VECTASTAIN ABC HRP kit, VECTOR Laboratories, Burlingame, CA, USA) and followed using DAB as detection reagent (VECTASTAIN DAB kit, VECTOR Laboratories, Burlingame, CA, USA) by using an automated immunohistochemistry autostainer (Ventana BenchMark Special Stains system, Ventana Medical System, AZ, USA). Negative controls were utilized without use of primary antibody. Normal human breast cancer tissues were used as positive controls. Immunohistochemistry staining studies were repeated three times.

The H&E slides and phosphor-NFκB immunostained with counterstain slides were reviewed by PCa-specialized pathologist and regionally matched to identify the adenocarcinoma areas and the normal and benign gland areas. The immunostained slides were scanned into digital images using Aperio Digital Pathology Slide Scanner. The intensity of immunostaining of phospho-NFκB in nuclei in PCa tissue sections was determined by using Leica Aperio Nuclear Algorithm (Leica Biosystems Inc, Buffalo Grove, IL, USA) to detect the average intensity of the entire tumor section. The percentage of Ki67-positive tumor cells was calculated also by using Leica Aperio Nuclear Algorithm. The Leica Aperio Nuclear Algorithm was developed by Leica Biosystems and has been validated for clinical utilization to analyze nuclear staining biomarkers such as estrogen receptor and Ki67 in cancer tissues.

### 4.6. Assessment of Primary Tumor and Metastasis

The primary PCa xenograft tumors observed in the prostate were grossly measured in area size using an electronic digital caliper. The formalin-fixed tumor with prostate tissue was then serially sliced into 2-millimeter-thick sections. All tissue sections were processed into paraffin-imbedded tissue sections on slides for microscopic examination and measurement. The H&E slides with primary tumor or metastatic tumor were scanned into digital images using Aperio Digital Pathology Slide Scanner, and the tumor volume was calculated by measuring tumor area size on slide ×2 mm thickness for each tissue section. The area size was measured using an Olympus CellSens imaging analysis software. Tumor necrotic area size was measured and deducted while calculating tumor volume. The calculated tumor volume was compared between vehicle IgG-treated and ALT-100-treated groups.

PCa cell invasion into prostate gland and prostate capsule were identified in each prostate tissue. All other organs were examined under microscopy to identify metastasis.

### 4.7. Statistic Analysis

Overall survival rate was analyzed by using log-rank (Mantel-Cox) test and Gehan–Breslow–Wilcoxon test. Student’s t test was used to compare tumor size, Ki67 proliferation index, and the percentage of mice with invasive or metastatic tumors between vehicle IgG-treated mice and ALT-100 mAb-treated PCa-SCID mice.

## 5. Conclusions

In summary, an eNAMPT-neutralizing therapy, via a humanized monoclonal antibody, appears to be an effective approach in preventing PCa growth, invasion, and metastasis. Therapeutic targeting of eNAMPT may potentially antagonize the switch of aggressive invasive disease in androgen deprivation therapy-resistant PCa and prevent PCa progression. While speculative, this is consistent with prior studies demonstrating that uncoupling the dynamic reciprocity that occurs in androgen-independent tumor interaction in the metastatic microenvironment results in a halt in progression to metastases. Clinical trials in men with PCa utilizing eNAMPT-neutralizing therapies, possibly selecting clinical trial subjects via further identified biomarkers or *NAMPT* genotypes, may prove to be an effective approach in halting PCa progression.

## Figures and Tables

**Table 1:** Summary of ALT-100 mAb therapeutic responses in DU145 and PC3 orthotopic xenograft mouse models.

PCa Orthotopic Xenograft		DU145			PC3		
Model Groups		IgG-Treated	ALT-100-Treated	*p* Value	IgG-Treated	ALT-100-Treated	*p* Value
Animal Number		10	10		10	10	
Age (wks)		8	8		10	10	
Prostate Tumor Size (mm^3^)		538.8 ± 170	165.2 ± 49.3	*p* < 0.05	1197 ± 518	418.03 ± 219	*p* < 0.05
Ki67 Proliferation Index		23.3 ± 1.9%	12.9 ± 1.1%	*p* < 0.05	27.3 ± 6.1%	13.3% ± 5.7%	*p* < 0.05
Local Invasion Rate (%)	Prostate Glands	100%	40%		100%	50%	
	Prostate Capsule	80%	10%		100%	40%	
Distant Meta-stasis Rate (%)	Lymph node	50%	0%		90%	50%	
	Intestine	40%	0%		60%	20%	
	Pancreas	30%	0%		50%	20%	
	Kidney	0%	0%		50%	10%	
	Liver	10%	0%		30%	30%	
	Lung	10%	0%		0%	0%	
	Bone	0%	0%		0%	0%	
Survival Rate at 12 wks		60%	100%	*p* < 0.05	30%	60%	*p* < 0.05

## Data Availability

The data presented in this study are available on request from the corresponding author. Data contained within the article and Supplementary Materials are publicly available.

## Supplementary Materials

The following are available online at https://www.mdpi.com/article/10.3390/ph14121322/s1, Figure S1: ALT-100 mAb Surface Plasmon Resonance (SPR), Figure S2: ALT-100 mAb Pharmacokinetics.
