# Supplementary material for: A Humanized Monoclonal Antibody Targeting Extracellular Nicotinamide Phosphoribosyltransferase Prevents Aggressive Prostate Cancer Progression
Source: Pharmaceuticals (Basel). 2021 Dec 17;14(12):1322. doi: 10.3390/ph14121322 (PMC8706080; doi:10.3390/ph14121322)
Supplement: Supplementary file 1 [file pharmaceuticals-14-01322-s001.zip › pharmaceuticals-1483015-supplementary.pdf]

Supplemental Data:

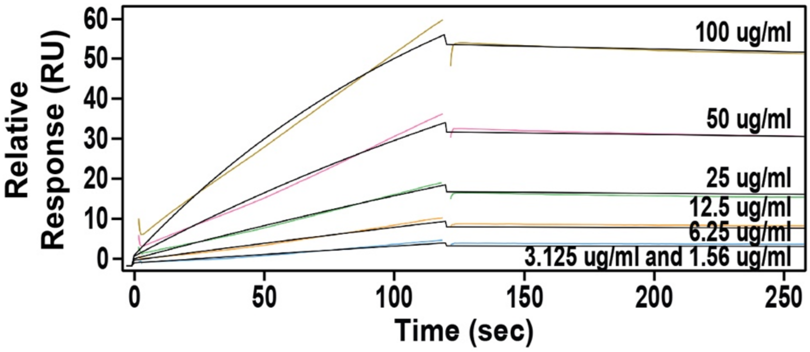

Supplemental Figure S1. ALT-100 mAb Surface Plasmon Resonance (SPR). ALT-100 mAb binding affinity was assessed using SPR with immobilization of rhNAMPT (His Tag peptide, MBL International). As seen, the equilibrium dissociation constant (K_d) is 6.33E-09 (M) with Rmax (RU) 99.1%.

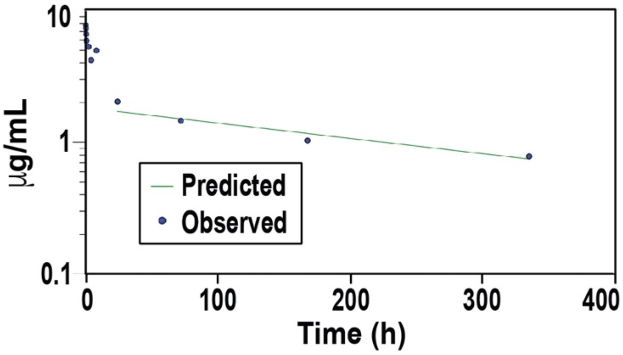

Supplemental Figure S2. ALT-100 mAb Pharmacokinetics. Shown are the average plasma concentration of ALT-100 mAb vs. time profiles in rats after a single IV dose at 100 µg/kg. Green line represents the predicted elimination phase, which was used to determine half-lives. Circles represent the average plasma concentration from six rats at each time point. The $T_{1/2}$ of ALT-100 mAb is 355.2 hours (14.8 days).
